# Prevalence of Diabetic Foot Ulcer and Associated Factors among Adult Diabetic Patients Who Attend the Diabetic Follow-Up Clinic at the University of Gondar Referral Hospital, North West Ethiopia, 2016: Institutional-Based Cross-Sectional Study

**DOI:** 10.1155/2017/2879249

**Published:** 2017-07-16

**Authors:** Tesfamichael G. Mariam, Abebaw Alemayehu, Eleni Tesfaye, Worku Mequannt, Kiber Temesgen, Fisseha Yetwale, Miteku Andualem Limenih

**Affiliations:** ^1^College of Medicine and Health Science, School of Nursing, University of Gondar, P.O. Box 196, Gondar, Ethiopia; ^2^College of Medicine and Health Science, Department of Midwifery, University of Gondar, P.O. Box 196, Gondar, Ethiopia

## Abstract

Diabetes mellitus is a metabolic disorder which is characterized by multiple long-term complications that affect almost every system in the body. Foot ulcers are one of the main complications of diabetes mellitus. However, there is limited evidence on the occurrence of foot ulcer and influencing factors in Ethiopia. An institutional-based cross-sectional study was conducted in Gondar University Hospital, Ethiopia, to investigate foot ulcer occurrence in diabetic patients. Systematic random sampling was used to select 279 study participants. Bivariate and multivariable logistic regression model was fitted to identify factors associated with diabetic foot ulcer. Odds ratio with 95% confidence interval was computed to determine the level of significance. Diabetic foot ulcer was found to be 13.6%. Rural residence [AOR = 2.57; 95% CI: 1.42, 5.93], type II diabetes mellitus [AOR = 2.58; 95% CI: 1.22, 6.45], overweight [AOR = 2.12; 95% CI: 1.15, 3.10], obesity [AOR = 2.65; 95% CI: 1.25, 5.83], poor foot self-care practice [AOR = 2.52; 95% CI: 1.21, 6.53], and neuropathy [AOR = 21.76; 95% CI: 8.43, 57.47] were factors associated with diabetic foot ulcer. Diabetic foot ulcer was found to be high. Provision of special emphasis for rural residence, decreasing excessive weight gain, managing neuropathy, and promoting foot self-care practice would decrease diabetic foot ulcer.

## 1. Introduction

Diabetes mellitus (DM) is one of the most important and common metabolic disorders affecting about 2–5% of the population in Europe and about 20% of the population in various other parts of the world [1]. The incidence of diabetes mellitus is increasing worldwide; by 2030, it will grow up to 366 million. This estimation occurred because of longer life expectancy and changing habits of diet [2].

Even though there are many complications affecting the person with diabetes, none are more devastating than those complications involving the foot [3]. Diabetic foot lesions have significant health and socioeconomic problems holding adverse effects on the quality of life of the patient and imposing a heavy economic burden on the patient's family [4].

Foot ulcers significantly contribute to morbidity and mortality of patients with diabetes mellitus. The diabetic patients with foot ulcers require long-term hospitalization and carry the risk of limb amputation [5].

Foot complications are common in diabetic patients and are considered one of the most expensive diabetes complications to treat [6]. People at greatest risk of ulceration can easily be identified by careful clinical examination of the feet during provision of health education about diabetes complication and during follow-up visits [7].

In developing countries, foot ulcers are one of the most feared and common complications of diabetes. They are a major cause of disability, morbidity, and mortality among diabetic patients, and it has been estimated that 15% of all people with diabetes will have an ulcer at some stage of their life [8].

The most important complications of diabetes mellitus are neuropathy and foot ulcer. Manifestations of complications range from simple to highly complex, including limb amputations and life-threatening infections [9].

Studies show that severity of diabetic foot ulcer is the strongest significant risk factor of amputation for diabetes patients [10]. In developed countries, one in every six people with diabetes will have an ulcer during their lifetime. The risk is even higher in developing countries [10].

Risk factors associated with the natural history of foot ulcer in diabetic patients include metabolic or biologic characteristics and the extrinsic characteristics which result from the patient's interaction with the environment. Peripheral neuropathy, peripheral vascular disease, and foot trauma were also reported risk factors in the pathophysiology of foot ulcer [11].

Diabetic foot ulcer is one of the long-term complications of diabetic mellitus with the life time risk up to 25%, yet many of the occurrences could be prevented [12]. Even though preventive strategies have been shown to be cost-effective, diabetic foot ulcers still occur frequently and are a challenge for the individual and for the health system [13]. The rapid increase of foot ulcer among people with diabetes requires solid epidemiological knowledge based on high-quality health care services and effective preventive strategies, which must be carefully tailored to the needs of specific groups [14]. Research indicates that diabetic foot ulcer is affected by several factors including patient age, educational status of the patient, weight of patient, type of diabetes mellitus, patient habits of foot self-care practice, and the presence of complicated peripheral neuropathy [10, 12–15]. However, the determinants of diabetic foot ulcer are not the same across different socioeconomic and demographic factors and progresses of disease within the institution. Thus, assessing factors affecting diabetic foot ulcer in different areas is very important to prevent the devastating effect of foot ulcer among diabetes patients. Therefore, this study aimed to assess diabetic foot ulcer and associated factors among adult diabetic patients attending the diabetic clinic at the University of Gondar Referral Hospital, North West Ethiopia. The finding of this study will help to decrease the occurrence of diabetic foot ulcer and its complication in the area.

## 2. Methods

### 2.1. Study Area

An institutional-based cross-sectional study was conducted from the 1st of March to the 30th of April, 2016, at Gondar University Hospital. The hospital is located in Gondar town, which is located 735 km to the northwest of Addis Ababa, the capital city of Ethiopia. In the hospital, there are fourteen different units which provide outpatient medical services to patients. Nearly 250,000 patients visit the outpatient clinics, and there are more than 21,000 admissions in this year. This hospital serves as a general hospital, a teaching hospital, and research center, and it serves as referral center for more than five million people. The hospital has one diabetic follow-up clinic, which serves around 5022 diabetic patients annually.

### 2.2. Source Population

This study includes all diabetes mellitus patients who attend the diabetic follow-up clinic at the University of Gondar Referral Hospital.

### 2.3. Study Population

This study includes all diabetes mellitus patients who attend the diabetic follow-up clinic at the University of Gondar Referral Hospital during the study period.

### 2.4. Inclusion Criteria

All adult diabetes mellitus patients, who attend the diabetic follow-up clinic at the University of Gondar Referral Hospital during the study period, were included in the study.

### 2.5. Exclusion Criteria

Diabetic patients who had traumatic ulcer due to car accident and those diabetic patients who were severely ill and unable to communicate throughout the study period were excluded.

### 2.6. Characteristics of Included Study Participants/Patients as Compared with the Excluded One

Those diabetic patients who had any diabetic-related ulcer were included. Rather than including all ulcers in diabetic patient, making specification on the type of occurrence of ulcer gives us a better understanding about the complication of diabetes on peripheral system. As a result, we want to exclude the ulcer which occurred due to accident.

### 2.7. Variable of the Study

#### 2.7.1. Dependent Variable

The dependent variable includes the presence of diabetic foot ulcer.

#### 2.7.2. Independent Variable

The independent variables are as follows.

1. Sociodemographic variables: age, sex, religion, ethnicity, marital status, educational status, area of residence, and average monthly income
2. Behavioural factors: smoking cigarette, alcohol consumption, and physical activity
3. Clinical factors: fasting blood sugar level, comorbidity (additional known disease), body mass index, history of ulceration, regular follow-up to the diabetic clinic, category of diabetes, peripheral vascular disease, neuropathy, and duration of diabetes mellitus
4. Foot self-care practice-related factors: characteristics of foot wear, footwear inspection, footwear practice, and foot washing.

### 2.8. Operational Definition

Diabetic foot ulcer is nontraumatic lesions of the skin (partial or full thickness) on the foot of a person who has diabetes mellitus.

#### 2.8.1. Knowledge about Diabetes

Knowledgeable are those participants who scored mean (16.8) and above from knowledge assessment questions.

#### 2.8.2. Foot Self-Care Practices

Those participants who scored mean (7) and above from foot self-care practice assessment questions are considered to do good foot self-care practices.

#### 2.8.3. Severity of Diabetic Foot Ulcer Based on Wagner's Classification

We have the following grades: Grade 0—no ulcer, but the foot is at risk for ulceration; Grade 1—superficial ulceration; Grade 2—ulcer with deep infection, but without involvement of the bone; Grade 3—ulcer with osteomyelitis; Grade 4—localized gangrene; Grade 5—gangrene of the whole foot.

#### 2.8.4. Body Mass Index (BMI)

It is calculated as the body weight of the individual patient divided to the square of their height; and we considered BMI ranges < 18.5 kg/m^2^ = underweight, BMI ranges 18.5–24.5 kg/m^2^ = normal range, BMI ranges from 24.5 to 30 kg/m^2^ = overweight and BMI > 30 kg/m^2^ = obese.

#### 2.8.5. Neuropathy

It was diagnosed if the patient had at least one manifestation from the following list of manifestations: burning pain, vibration from the skin, gradual numbness, freezing, extreme sensitive to touch, muscle weakness, and lack of coordination.

#### 2.8.6. Measurement of Diabetes Mellitus

Fasting blood sugar level on each individual patient was done and fasting blood sugar level greater than 125 mg/dl was considered as diabetic.

#### 2.8.7. Controlled Diabetes Mellitus

If the fasting blood glucose level was between 100 and 125 mg/dl, it was considered “controlled.”

#### 2.8.8. Peripheral Vascular Disease

It is an arterial and vein disease at the peripheral region, which often occurs in diabetic patient. It was diagnosed if the diabetic patient had at least one of the following manifestations: painful cramping in their hip, muscle cramping after movement, leg numbness, change the colour of the legs, shiny skin on the leg, sores on the toes, feet or legs that will not heal, and erectile dysfunction.

### 2.9. Sample Size Calculation and Sampling Procedure

Single population proportion formula was used to calculate the required sample size considering the following assumptions: prevalence of diabetic foot ulcer 12% [14], 95% confidence level, and 4% margin of error (absolute level of precision).

(1) $$\begin{array}{c} n=\frac{{\left(z\alpha /2\right)}^{2}p\left(1-p\right)}{d^{2}}=\frac{{\left(1.96\right)}^{2}0.12\left(0.88\right)}{{\left(0.04\right)}^{2}}=253, \end{array}$$

with the assumptions being as follows: *n* is required sample size, *p* is prevalence of adult diabetic foot ulcer (12%), the rate found in recent research at a similar referral hospital in Ethiopia [14], *Z* is standardized normal distribution value at the 95% CI: 1.96, and *d* is the margin of error of 4. The low design effect was used to increase sample size. The final sample size was adjusted by using the probability of 10% nonresponse rate, and the total sample size was adjusted to be equal to 279 participants.

#### 2.9.1. Sampling Procedure

A systematic random sampling technique was used to select the study participants. We identified the average patient flow at the diabetic follow-up clinic in the study area. Based on the previous year's data, there were 5022 diabetic patients per year who had diabetic clinic follow-up. Then we divided this total number of patients to twelve months, resulting in an average of 418.5 diabetic patients per month and an average of 837 patients per two months. In order to get the study unit, we used the principles of systematic random sampling approach. Since our study period was two months, we considered the previous two-month diabetic patient flow as the source population. Then we divided to the total required sample size to get the interval/fraction (*K*).

(2) $$\begin{array}{c} K=\frac{N}{n}=\left(\frac{837}{279}=3\right). \end{array}$$

Then to start the interview, we had selected by using the lottery method from patient one to patient three. As a result, patient three was selected randomly using the lottery method. Then the interview started from the third patient attending the clinic and continued by recruiting every third patient based on their sequence of exit after check-up, up to the required 279 participants, which is fulfilled during the study period.

### 2.10. Data Collection and Analysis

Data were collected using a structured and pretested questionnaire via face-to-face interview, a record review, and direct observation of patient. The questionnaire was prepared in English and then translated to local language (Amharic) then back to English to keep its consistency. Three BSc nurses and one MSc nurse were involved in the data collection process. One-day training was given for both data collectors and supervisor. All adult diabetes mellitus patients, who attended diabetic the follow-up clinic at the University of Gondar Hospital during the study period, were included in this study. Diabetic foot ulcer was measured as nontraumatic lesions of the skin (partial or full thickness) on the foot of a person who has diabetes mellitus.

Data were entered using EPI-INFO version 3.5.3 and exported to SPSS statistical software for further analysis. Descriptive statistics were carried out to characterize the study population using different variables. Both bivariate and multiple logistic regressions were used to identify associated factors. Variables having *p* value ≤0.2 in the bivariate analyses were fitted into multiple logistic regression models to control the effects of confounding. Crude and adjusted odds ratio with their 95% CI was calculated to determine the presence of association. A variable with a *p* value of 0.05 was considered a significant predictor.

### 2.11. Ethical Considerations

Ethical clearance was obtained from the Ethical Review Committee of the School of Nursing, University of Gondar. An official letter of cooperation was written to the University of Gondar Referral Hospital administration. After explaining the purpose of the study, written informed consent was obtained from each of the study participant. Participants were also informed that participation was on a voluntary basis and that they could withdraw at any time, for any reason. Personal identifiers were not included in the written questionnaires to ensure participants' confidentiality.

## 3. Results

### 3.1. Sociodemographic Factors

A total of 279 adult diabetic patients who had diabetic follow-up were involved in the study. We did not get excluded study participants based on exclusion criteria. From the total number of participants involved in the study, 154 (55.2%) were males and 125 (44.8%) were females. The mean age of participants was 49.8 with SD ± 15.6 years. One hundred ninety (68.1%) were married. Regarding their educational status, 46 (16.5%) had secondary education and above. Ninety-nine (35.5%) participants came from rural area. Two hundred forty-six, (88.2%) participants were Orthodox Christians on religious status (Table 1).

### 3.2. Behavioural Factors

Eighteen (6.5%) of the study participants were smokers. Among those who smoke, 17 (94.4%) of them were daily smokers. Ninety-one (32.6%) study participants were alcohol drinkers. Among those who drink alcohol, 54 (59.3%) of study participants were daily alcoholic drinkers. Regarding involvement in physical exercise, 228 (81.7%) of the participants claimed that they engaged in different physical exercises. The type of exercise reported by about 158 (69.3%) of study participants was movement during routine working activity. Two hundred twenty-seven participants (99.5%) wear shoes.

### 3.3. Clinical Factors

Among the total 279 study participants, 251 (90%) had regular follow-up to the diabetic clinic of Gondar University Hospital and 169 (60.6%) of them had type 2 diabetes mellitus. A majority of the study participants have a BMI between18 and 24.5 kg/m^2^. The mean fasting blood glucose level among diabetic patients with foot ulcer was 128.58 mg/dl. One hundred and eight participants (38.7%) were diabetic for more than 6 years. One hundred and two (36.6) participants had poorly controlled blood glucose levels. About 70 (25.1%) of the participants had chronic health problems or comorbidity with other diseases, and among these, 50 (71.4) participants were hypertensive. Forty-six (16.5) study participants had sensation loss to vibration. Peripheral vascular disease was detected in 27 (9.7) participants and 28 (10%) had peripheral neuropathy. Similarly, 32 (11.5%) of the study population had callus (Table 2).

### 3.4. Knowledge on DM and Practice on Foot Self-Care

One hundred sixty-eight (60.2%) study participants were knowledgeable about diabetes, and the remaining was not knowledgeable. Regarding diabetic foot self-care practice, good foot self-care practice was observed among 102 (36.6%) participants and the remaining 177 (63.4%) study participants poorly practiced foot self-care.

### 3.5. Prevalence of Diabetic Foot Ulcer

**Figure 1:**
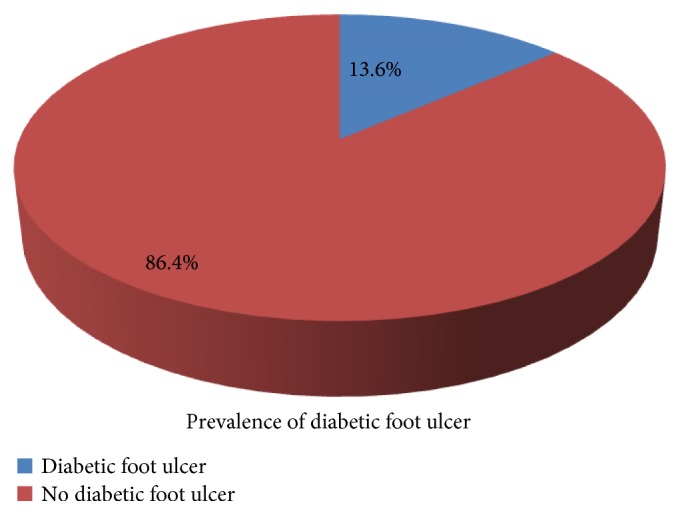
Prevalence of diabetic foot ulcer among adult diabetic patients in Gondar University Referral Hospital, Diabetic Clinic, 2016 (*n* = 279).

Among 279 study participants in the diabetic clinic of Gondar University Referral Hospital, thirty-eight (13.6%) patients had developed foot ulcer (Figure 1).

### 3.6. Factors Associated with Diabetic Foot Ulcer

Residence [AOR = 2.57; 95% CI: 1.42, 5.93], types of diabetes mellitus [AOR = 2.58; 95% CI: 1.22, 6.45], overweight [AOR = 2.12; 95% CI: 1.15, 3.10], obesity [AOR = 2.65; 95% CI: 1.25, 5.83], foot self-care practice [AOR = 2.52; 95% CI: 1.21, 6.53], and neuropathy [AOR = 21.76; 95% CI: 8.43, 57.47] were found to be significantly associated with diabetic foot ulcer in multivariable logistic regression analysis.

Those diabetic patients who lived in the rural area were 2.57 times more likely to develop diabetic foot ulcer than those who lived in the urban area [AOR = 2.57; 95% CI: 1.42, 5.93]. Diabetic patients who had type II DM were 2.58 times more likely to develop diabetic foot ulcer than those who had type I DM [AOR = 2.58; 95% CI: 1.22, 6.45]. Overweight diabetic patients were 2.12 times more likely to develop diabetic foot ulcer as compared to diabetic patients with normal weight [AOR = 2.12; 95% CI: 1.15, 3.10]. Obese diabetic patients were 2.65 times more likely to develop diabetic foot ulcer as compared to diabetic patients with normal body mass index [AOR = 2.65; 95% CI: 1.25, 5.83]. In addition, those diabetic patients who had not practiced foot self-care were 2.52 times more likely to develop diabetic foot ulcer than those diabetic patients who had practiced foot self-care [AOR = 2.52; 95% CI: 1.21, 6.53]. Further, those diabetic patients who had neuropathy were 21.7 times more likely to develop diabetic foot ulcer as compared to those diabetic patients without neuropathy [AOR = 21.76; 95% CI: 8.43, 57.47] (Table 3).

## 4. Discussion

This study result revealed that the prevalence of diabetic foot ulcer among diabetic patients who attend diabetic clinic follow-up was13.6% (95% CI: 9.3, 17.2).This finding is in line with the studies done with diabetic patients in Arbaminch, Ethiopia (14.8%), and Mekele, Ethiopia (12%) [12, 15].

However, this study finding was lower than the study conducted in Addis Ababa, Ethiopia, and Nigeria which found diabetic food ulcer prevalence to be 31.1% and 41.1%, respectively [8, 16]. This variation might be due to difference in sample size or due to differences in geographical location of the studies as well as sociocultural variation of the study participants.

On the other hand, the finding of the current study is higher when compared to a study conducted in Kenya which was stated as the prevalence of diabetic foot ulcer among diabetic patients was 4.6% [5]. The possible explanation for this difference could be due to difference in knowledge-related diabetic foot self-care practice, knowledge on diabetes mellitus, and also possibly due to difference on health-seeking behaviour practice between the two study populations.

Those diabetic patients who lived in the rural area were 2.57 times more likely to develop diabetic foot ulcers than diabetic patients from the urban area [AOR = 2.57; 95% CI: 1.42, 5.93]. This finding is in line with the studies conducted in Arbaminch, Ethiopia; Mekele, Ethiopia; and Colombia [5, 12, 17]. Diabetic patients who live in rural areas of Ethiopia often spent most of their time in farm area or outdoors and may be subjected to rodent bites of their feet. Bites to the feet of patients with diabetes can lead to the development of ulceration due to poor wound healing process and less opportunity for health care service for it. Another possible explanation might be those diabetic patients who lived in the rural area had poor awareness about personal hygiene and foot self-care practice, and they often walk with bare feet. This may expose their feet to harm and lead to the development of foot ulcer.

The finding of this study showed that overweight diabetic patients were 2.1 times more likely to develop diabetic foot ulcer as compared with those who had a normal weight [AOR = 2.1; 95% CI: 1.15, 3.10]. Further, these obese diabetic patients were 2.65 times more likely to develop diabetic foot ulcer as compared to those diabetic patients who were not obese [AOR = 2.65; 95% CI: 1.25, 5.83]. This is consistent with the studies conducted in Ethiopia, Kenya, and Malaysia [5, 8, 16]. The possible reason could be due to the presence of higher foot pressure in those heavily weighed and with higher body mass index (BMI) diabetic patients as well obesity and overweight might decrease intensively the normal blood circulation pattern at the lower extremities; as a result, this might lead them to develop diabetic foot ulcer.

Type of diabetes mellitus was one of the strongest predictors of diabetic foot ulcer occurrence. Those diabetic patients who had type II diabetes mellitus were 2.58 times more likely to develop diabetic foot ulcer than those who had type I DM [AOR = 2.58; 95% CI: 1.22, 6.45]. This finding is consistent with the studies conducted in Nigeria, Egypt, and Asia [2, 16, 18] which indicated type II diabetes mellitus was significantly associated with the occurrence of diabetic foot ulcer. The possible explanation could be in type II diabetic patients; there are related complications of the disease, such as mechanical changes in the conformation of the bony architecture of the foot, peripheral neuropathy, and atherosclerotic peripheral arterial disease; as a result, the patient may have less tissue epithelisation, consumption of oxygen, nutrient transportation, and cell detoxification resulting in ulceration in the extremities.

Having neuropathy was another variable which had a strong association with foot ulcers in diabetic patients. Diabetic patients who had neuropathy were 21.7 times more likely to develop diabetic foot ulcer as compared to diabetic patients without neuropathy [AOR = 21.76; 95% CI: 8.43, 57.47]. This result is consistent with the studies conducted in Tanzania, Jordan, and Egypt [1, 2, 19]. Diabetic patients with high blood glucose level are exposed to microvascular complication and neuropathy, and the occurrence of neuropathy may increase the risk for foot ulceration due to increased pressure load and shearing forces. In addition to this, those chronic diabetes patients at early postpartum period who used combined hormonal contraceptive methods may have an effect on the risk of deep venous thrombosis, which also has the risk of neuropathy and may aggravate the occurrence of diabetic foot ulcer [20].

Diabetic foot ulcer was strongly influenced with lack of foot self-care practice. Those diabetic patients who had not practiced foot self-care were 2.52 times more likely to develop diabetic foot ulcer than those diabetic patients who had practiced foot self-care [AOR = 2.52; 95% CI: 1.21, 6.53]. This finding is similar with the studies conducted in Arbaminch, Ethiopia; Mekele, Ethiopia; Kenya; and India [4, 5, 12, 15]. Practicing foot self-care could reduce the development of diabetic foot ulcer due to the benefits of washing their own feet regularly, drying appropriately after washing, daily evaluation of their foot status, and/or facilitating circulation and early management of any abnormality that may occur on the foot.

### 4.1. Limitation of the Study

There might be recall bias or reporting bias regarding the contributing factors, such as alcohol use or exercise frequency. Further, the cross-sectional nature of the study does not confirm the definitive cause and effect relation.

## 5. Conclusion

The prevalence of diabetic foot ulcer among diabetic patients in Gondar University Referral Hospital was found to be high. Residence, higher BMI (overweight and obesity), types of diabetes, neuropathy, and foot self-care practice were factors significantly associated with diabetic foot ulcer. The health care providers are recommended to enhance preventive measures in the reduction of foot ulcer through promoting foot self-care practice, giving special emphasis during follow-up of patients who came from rural areas, educating the patient to reduce overweight gain, and managing the neuropathy thoroughly in order to decrease the occurrence of diabetic foot ulcer.

## Figures and Tables

**Table 1:** Sociodemographic characteristics of respondents in Gondar University Referral Hospital, Northwest, Ethiopia 2016 (*n* = 279).

Variable	Frequency	Percent
*Age*		
18–27	36	12.9
28–37	30	10.8
38–47	45	16.2
48–57	65	23.2
58–67	62	22.2
>68	41	14.7
*Marital status*		
Single	51	18.3
Married	190	68.1
Separated	7	2.5
Divorced	14	5.0
Widowed	17	6.1
*Occupation*		
Housewife	82	29.4
Merchant	58	20.8
Farmer	62	22.2
Government employed	44	15.8
Daily laborer	33	11.8
*Level of education*		
Unable to read	137	49.1
Can read and write	72	25.8
Primary education	24	8.6
Secondary education and above	46	16.5
*Residence*		
Urban	180	64.5
Rural	99	35.5
*Monthly income*		
<25$	65	23.3
25$–50$	143	51.3
50$–100$	58	20.7
>100$	13	4.7

**Table 2:** Clinical factors of respondents in Gondar University Referral Hospital, Northwest, Ethiopia, 2016 (*n* = 279).

Variable	Frequency	Percent
*Previous history of diabetic foot ulcer*		
Yes	24	8.6
No	255	91.4
*Diabetic medication currently*		
Oral hypoglycemic	148	53.0
Insulin	131	47.0
*Special prescribed diet*		
Yes	273	97.8
No	6	2.2
*Duration of DM*		
<5 years	155	55.6
6–10 years	108	38.7
11–15 years	16	5.7
*Blood glucose level controlled by current medication*		
Good controlled	177	63.4
Poorly controlled	102	36.6
*Regular follow-up*		
Yes	251	90.0
No	28	10.0
*Category of DM*		
Type one	110	39.4
Type two	169	60.6
*Additional known disease*		
Yes	70	25.1
No	209	74.9
*Types of additional disease (n* = 70)		
Hypertension	50	71.4
Renal disease	16	22.9
Asthma	2	2.9
Heart disease	2	2.9
*Callus*		
Yes	32	11.5
No	247	88.5
*Sensory loss to vibration*		
Yes	46	16.5
No	233	83.5
*Peripheral vascular disease*		
Yes	27	9.7
No	252	90.3
*Neuropathy*		
Yes	28	10.0
No	251	90.0
*Body mass index*		
<18	**21**	**7.5**
18–24.49	**137**	**49.1**
24.5–29.5	**65**	**23.3**
>29.5	**56**	**20.1**

**Table 3:** Bivariate and multivariate analysis of factors associated with diabetic foot ulcer among adult diabetic patients in Gondar Referral Hospital, Diabetic Clinic, 2016 (*n* = 279).

Variables	DM foot ulcer		COR (95% CI)	AOR (95% CI)
	Yes	No		
*Sex*				
Male	27	127	2.20 (1.16, 4.64)	^∗∗^
Female	11	114	1	
*Educational status*				
Unable to read and write	22	115	4.27 (1.23, 14.81)	^∗∗^
Able to read and write	13	59	4.92 (1.34, 18.12)	^∗∗^
Formal education	3	67	1	
*Residence*				
Urban	14	166	1	**1**
Rural	24	75	3.79 (1.86, 7.74)	**2.57 (1.42, 5.93)**
*Current smoking*				
Yes	8	10	6.16 (2.26, 16.82)	^∗∗^
No	30	231	1	
*Previous history of smoking*				
Yes	9	10	7.17 (2.69, 19.10)	^∗∗^
No	29	231	1	
*Currently drink alcohol*				
Yes	17	74	1.83 (0.91, 3.66)	^∗∗^
No	21	167	1	
*History of alcoholic drink*				
Yes	17	74	1.83 (0.91, 3.66)	^∗∗^
No	21	167	1	
*Physical activity*				
Yes	22	206	1	
No	16	35	4.28 (2.05, 8.94)	^∗∗^
*Previous history of diabetic foot ulcer*				
Yes	8	16	3.75 (1.48, 9.51)	^∗∗^
No	30	225	1	
*Specially prescribed diet*				
Yes	35	238	1	
No	3	3	6.8 (1.32, 35.0)	^∗∗^
*Regular follow-up*				
Yes	18	233	1	
No	20	8	32.4 (12.5, 83.7)	^∗∗^
*Type of diabetes mellitus*				
Type one	7	103	1	**1**
Type two	31	138	3.31 (1.40, 7.80)	**2.58 (1.22, 6.45)**
*Callus of foot*				
Yes	19	13	17.54 (7.52, 40.89)	^∗∗^
No	19	228	1	
*Peripheral vascular disease*				
Yes	16	11	15.21 (6.29, 36.80)	^∗∗^
No	22	230	1	
*Neuropathy*				
Yes	20	8	32.36 (12.52, 83.66)	**21.76 (8.43, 57.47)**
No	18	233	1	**1**
*Body mass index*				
<24.5	14	143	1	**1**
24.5–29.5	18	78	2.36 (1.11, 4.10)	**2.12 (1.15,3.10)**
>29.5	6	20	3.06 (1.06, 8.89)	**2.65 (1.25, 5.83)**
*Knowledge on diabetes mellitus*				
Not knowledgeable	25	86	3.47 (1.69, 7.12)	^∗∗^
Knowledgeable	13	155	1	
*Foot self-care practice*				
Good practice	6	96	1	**1**
Poor practice	32	145	3.53 (1.42, 8.77)	**2.52 (1.21,6.53)**

Note: ^∗∗^not statistically associated with diabetic foot ulcer in multivariate logistic regression analysis with *p* value <0.05 at 95% CI.
